# Dr. Seneca D. Powell

**Published:** 1907-09

**Authors:** 


### OBITUARY.

Dr. Seneca D. Powell, of New York, died at his country home, Greenwich, Conn., August 24, 1907, in the sixtieth year of his age. He was born in Alabama, January 5, 1848, and at the outbreak of the civil war, while serving as a cadet in the University of Alabama, he left college to join the confederate army. After serving until the close of the war he entered the medical college of the University of Virginia from which he graduated in 1869, and in 1870 he received the degree of doctor in medicine from the New York University.

Dr. Powell served as interne at Bellevue Hospital and became a lecturer on surgery at the N. Y. Post-Graduate Medical School in 1883. Four years later he was made professor of surgery and for a number of years was secretary of the faculty. He was one of the vice-presidents of the first Pan-American Medical Congress (1893) and in 1898 was president of the Medical Society of the State of New York. His death was attributed to experiments relating to alcohol as an antidote for carbolic acid poisoning, on which he published several monographs.

